# Evaluation of professional course teaching behavior and self-reported professional competence in medical students: the effects of professional identity evaluation and campus environment satisfaction

**DOI:** 10.1186/s12909-026-09096-9

**Published:** 2026-04-01

**Authors:** Ting Zhang, Feng Feng Chen, Zhongxian Duan, Henggui Liang, Wei Wei

**Affiliations:** 1 School of Management, Guizhou University, Guiyang, Guizhou 550025 China; 2 School/Hospital of Stomatology, Guizhou Medical University, Guiyang, Guizhou 550004 China; 3 Guizhou Education Department, Guiyang, Guizhou 550081 China; 4 POWERCHINA Guizhou Electric Power Engineering Co., Ltd., Guiyang, Guizhou 550000 China

**Keywords:** Teaching behavior evaluation, Self-reported professional competence, Professional identity evaluation, Campus environment satisfaction, Medical students

## Abstract

**Objective:**

This study examines the association between professional course teachers' teaching behavior and medical students' self-reported professional competence, with a particular focus on the mediating role of professional identity evaluation and the moderating role of campus environment satisfaction.

**Methods:**

A cross-sectional survey design was employed, recruiting 430 full-time undergraduate students from a medical university in Guizhou Province, China. Data were collected via self-reported questionnaires measuring four core variables—evaluation of teaching behavior, professional identity evaluation, campus environment satisfaction, and self-reported professional competence—using 5-point Likert scales. Mediation and moderation analyses were conducted using the PROCESS macro for SPSS.

**Results:**

(1) Teaching behavior evaluation was significantly and positively associated with self-reported professional competence; (2) Professional identity evaluation partially mediated this relationship; and (3) Campus environment satisfaction significantly moderated the association between professional identity evaluation and self-reported professional competence, such that the positive relationship was stronger at higher levels of campus environment satisfaction.

**Conclusions:**

These findings offer theoretical insights and practical implications for optimizing teaching behaviors and enhancing the cultivation of professional competence among medical students.

**Supplementary Information:**

The online version contains supplementary material available at 10.1186/s12909-026-09096-9.

## Introduction

In competency-based medical education, cultivating medical students' professional competence represents a central mission [1], as it directly shapes the quality of future healthcare delivery and public trust in the medical profession [2]. Professional competence has evolved beyond clinical skills and theoretical knowledge to encompass an integrated construct comprising clinical reasoning, communication and collaboration, critical thinking, professionalism, ethical awareness, and lifelong learning capabilities—all essential for addressing complex challenges in modern healthcare systems. Given the pivotal role of professional course teachers in knowledge transmission, value formation, and role modeling, their teaching behaviors are widely recognized as critical inputs influencing students' learning experiences and developmental outcomes. Understanding how students' evaluations of professional course teachers' teaching behaviors relate to their self-reported professional competence therefore holds substantial theoretical significance and practical urgency for improving medical education quality and optimizing talent development [3, 4].

Despite growing literature on teacher behaviors and student outcomes in medical education, existing studies exhibit three critical limitations. First, most research focuses on direct associations between teacher behaviors and academic performance, learning engagement, or satisfaction [5, 6], while few systematically examine their long-term impacts on the formation and development of professional competence as a multifaceted competency outcome. Second, existing investigations predominantly adopt a direct-effects paradigm [7], overlooking the intervening psychological mechanisms that explain how teaching behaviors influence professional competence—leaving the “black box” of underlying processes largely unexplored. Third, few studies examine the boundary conditions under which such relationships are strengthened or weakened, particularly the potential moderating role of perceived campus environmental support. Collectively, these gaps constrain comprehensive understanding of how to translate effective teaching into sustainable improvements in medical students' professional competence.

To address these limitations, this study constructs a moderated mediation model grounded in Social Cognitive Theory [8], Self-Determination Theory [9] and Ecological Systems Theory [10]. The model positions professional identity evaluation as a mediator and campus environment satisfaction as a moderator to elucidate the complex association between professional course teachers' teaching behavior evaluation and medical students' self-reported professional competence. By empirically testing this integrated framework, this study aims to achieve three objectives: (1) clarify the direct association between teaching behavior evaluation and self-reported professional competence; (2) verify the mediating role of professional identity evaluation in transmitting the effects of teaching behaviors; and (3) reveal the moderating effect of campus environment satisfaction on the relationship between professional identity evaluation and self-reported professional competence.

This research contributes to the literature in three meaningful ways. Theoretically, it integrates individual cognitive and environmental factors into a unified model, enriching theoretical explanations of how teaching inputs shape competency outcomes in medical education. Practically, it provides empirical evidence to inform medical educators and administrators in designing targeted teaching improvements, strengthening professional identity development, and optimizing campus support systems to enhance students' professional competence. Methodologically, it employs a moderated mediation design to capture both mediating mechanisms and contextual boundaries, offering a rigorous analytical template for future educational psychology research in health professions education.

### The relationship between evaluation of professional course teaching behavior and professional identity evaluation

Social Cognitive Theory posits that individuals acquire knowledge, values, and behavioral norms through observational learning and social interaction, with significant others such as teachers serving as critical role models [11]. In medical education, professional course teachers function not merely as transmitters of specialized knowledge but as carriers of professional values and ethical standards [12]. Their teaching enthusiasm, interactive approaches, timely feedback, and professional integrity profoundly influence students' cognitive and affective perceptions of the medical profession [12]. Concurrently, Self-Determination Theory emphasizes that supportive teaching behaviors satisfy students' three fundamental psychological needs—autonomy, competence, and relatedness—thereby facilitating the internalization of professional values and the consolidation of professional identity.

Empirical research has confirmed that positive teaching behaviors significantly predict medical students' professional identity [13]. For instance, teachers' role modeling, patient-centered communication, and supportive feedback substantially enhance students' identification with their future profession [14]. Clinical teachers' dedication and sense of professional responsibility have also been found to strongly predict medical students' professional identity levels [15]. However, few studies have examined, within the context of professional coursework, how students' subjective evaluations of teaching behavior influence their professional identity evaluation.

- H1: Evaluation of professional course teaching behavior is significantly and positively associated with professional identity evaluation.

### The relationship between professional identity evaluation and self-reported professional competence

Professional identity refers to individuals' recognition, acceptance of, and emotional commitment to their professional roles, values, and norms [16]. According to Career Construction Theory and Social Identity Theory, strong professional identity motivates individuals to internalize professional standards, engage in learning activities, and strive to meet role expectations [17]. For medical students, higher professional identity is accompanied by enhanced learning persistence, more proactive participation in clinical practice, and stronger motivation to develop comprehensive professional competence [18, 19].

Existing evidence indicates that professional identity serves as a crucial motivational driver of competence development [20]. Students with clearer professional identities demonstrate higher levels of self-reported clinical competence, communication skills, and professional responsibility. Nevertheless, few studies have directly examined the relationship between “professional identity evaluation” as a cognitive-affective judgment and multidimensional “self-reported professional competence.”

- H2: Professional identity evaluation is significantly and positively associated with medical students' self-reported professional competence.

### The relationship between evaluation of professional course teaching behavior and self-reported professional competence

Both the Educational Production Function Theory and Social Learning Theory emphasize that teacher behavior constitutes a critical determinant of student learning outcomes and competence development [21]. In medical professional education, well-structured instruction, clinically oriented content, interactive teaching methods, and ethical modeling directly facilitate students' mastery of professional knowledge, clinical skills, and core competencies [22]. Through observation and imitation of teachers' professional behaviors, students gradually internalize the attitudes and competencies required of qualified medical professionals.

Although prior research has linked effective teaching to learning outcomes, few studies have examined the direct relationship between students' evaluations of professional course teaching behavior and overall self-reported professional competence encompassing cognitive, behavioral, and affective domains.

- H3: Evaluation of professional course teaching behavior is significantly and positively associated with medical students' self-reported professional competence.

### The mediating role of professional identity evaluation

Mediation models elucidate how independent variables influence dependent variables through intermediate variables. Grounded in Social Cognitive Theory and the Cognitive-Affective Personality System Theory, teachers' teaching behaviors may not only exert direct effects on professional competence but also operate indirectly by strengthening professional identity as a core motivational resource [23]. Supportive teaching enhances professional identity, which in turn drives students to pursue higher levels of professional competence [24].

Recent research supports the mediating role of professional identity between educational inputs and competence outcomes [25]. However, no empirical study has yet examined the mediating function of professional identity evaluation in the pathway from teaching behavior evaluation to self-reported professional competence.

- H4: Professional identity evaluation partially mediates the relationship between evaluation of professional course teaching behavior and medical students' self-reported professional competence.

### The moderating role of campus environment satisfaction

Ecological Systems Theory posits that individual development is embedded within and shaped by environmental systems [10]. Campus environment satisfaction—defined as students' subjective evaluations of academic resources, institutional support, interpersonal climate, and learning conditions—represents a critical microsystem factor influencing individual development [26]. A positive and satisfying campus environment can provide resources, encouragement, and feedback that facilitate the translation of professional identity into professional competence, thereby strengthening this relationship; conversely, a deficient environment may attenuate this association [27, 28].

Empirical evidence demonstrates that campus environment and institutional support moderate relationships between educational processes and outcomes [26]. Yet no study has examined whether campus environment satisfaction moderates the relationship between professional identity evaluation and self-reported professional competence.

- H5: Campus environment satisfaction positively moderates the relationship between professional identity evaluation and self-reported professional competence, such that this relationship is stronger at higher levels of satisfaction.

**Fig. 1:**
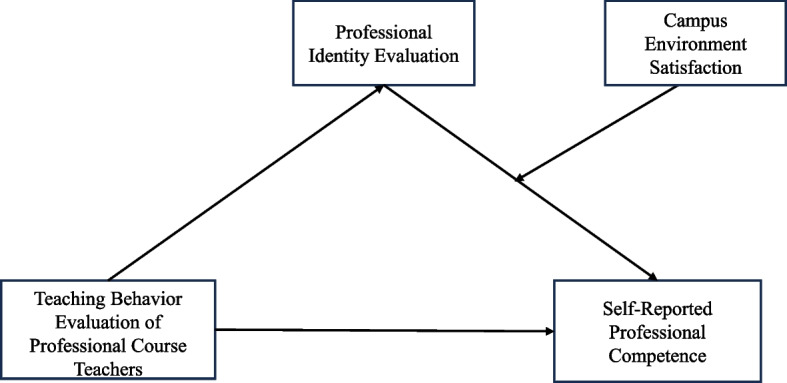
Theoretical model

This study explores the association mechanism through which the evaluation of professional course teachers' teaching behavior is associated with medical students' self-reported Professional Competence by constructing mediating and moderating models, aiming to provide new insights into improving the cultivation of medical students' self-reported Professional Competence. The theoretical model is illustrated in Fig. 1.

## Method

### Data source and sample characteristics

The sample population for this study consisted of full-time undergraduate students from a medical university in Guizhou. Data collection was performed by MyCOS Data Co., Ltd., a renowned third-party data consulting and assessment agency in China, from May to August 2022. A stratified cluster sampling method was adopted to ensure sample representativeness and reliability.

In the initial stage (May–June 2022), the research team collaborated with a medical university that provided basic information on medical students and course schedules. MyCOS designed and optimized the survey questionnaire based on this information. The questionnaire covered four core variables: evaluation of professional course teachers' teaching behavior, medical students' self-reported Professional Competence, professional identity evaluation, and campus environment satisfaction, measured using a Likert 5-point scale.

During the data collection phase (July–August 2022), the research team, coordinated by the university, organized medical students to complete the questionnaire online. To ensure data quality, the survey was administered anonymously, with MyCOS providing technical support for real-time monitoring of data completeness.

A total of 664 questionnaires were distributed (targeting students from freshman to senior years, excluding fifth-year interns), with 621 valid questionnaires returned, yielding a response rate of 93.52%. After data cleaning (including removing incomplete, patterned responses and outliers), 430 valid questionnaires were obtained, resulting in an effective rate of 69.24%. The gender distribution of the sample was 36.28% male (156) and 63.72% female (274) participants. The grade distribution was as follows: 20.23% freshmen (87), 29.07% sophomores (125), 23.95% juniors (103), and 26.75% seniors (115). The gender and grade distribution of the sample was generally consistent with the overall structure of medical students at the university, indicating good representativeness.

### Research instruments

The scale design of this study was primarily informed by Alexander Astin's “Input-Environment-Output” (I-E-O) model of student development [29] and High-Impact Practices (HIPs) [30], with survey content selected in alignment with current priorities in Chinese higher education, including institutional evaluation and professional accreditation. The scale items were adapted from established student engagement surveys, including the National Survey of Student Engagement (NSSE) [31], the Chinese version of NSSE (Tsinghua University), the College Student Experiences Questionnaire (CSEQ) [32], the Student Experience in the Research University (SERU) Survey, and the College Student Survey (Peking University). These instruments were localized and adapted to reflect the specific context of medical education in China through a rigorous process involving translation, back-translation, expert review, and pilot testing, resulting in the final formal questionnaire. All scales employed a 5-point Likert scale ranging from “1 = Strongly Disagree” to “5 = Strongly Agree,” with higher scores indicating higher levels of the corresponding variables.

The evaluation of Confirmatory Factor Analysis (CFA) fit indices in this study prioritized core fit indicators while treating secondary indicators as supplementary: the Normed Fit Index (NFI) and Comparative Fit Index (CFI) ≥ 0.85 served as acceptable thresholds for structural validity; internal consistency reliability (Cronbach's α) and Composite Reliability (CR) ≥ 0.7 were established as reliability standards; and Average Variance Extracted (AVE) ≥ 0.5 was set as the ideal threshold for convergent validity. The chi-square to degrees of freedom ratio (χ^2^/df) and Root Mean Square Error of Approximation (RMSEA) for the scales in this study did not reach ideal thresholds; these indices were therefore treated as reference only and not used independently for validity determination. The deviation in these results was primarily attributable to two factors: the specific context of medical education research and sample heterogeneity. On one hand, the scale items were closely aligned with the core characteristics of medical professional training, encompassing medical education-specific contexts such as clinical case-based teaching, medical professional competency, and specialized laboratory and clinical training resources. The strong professional specificity of these items resulted in pronounced differentiation among evaluation dimensions, which objectively affected absolute model fit indices. On the other hand, the survey sample comprised medical students across different academic years, with significant hierarchical differences in professional learning experiences, clinical practice exposure, and professional cognition levels. These differences led to substantial heterogeneity in subjective perceptions of teaching behaviors and campus environment across evaluation dimensions, further influencing model fit indices.

The overall reliability and validity of the questionnaire were satisfactory, with item statements closely reflecting the learning and living experiences of medical students. The specific information for the four core scales is as follows:

#### Evaluation scale for teaching behaviors of professional course teachers

This scale measures medical students' overall evaluation of teaching behaviors exhibited by instructors in professional courses. It was developed based on relevant dimensions of teaching behaviors from the aforementioned established domestic and international surveys, while incorporating the core characteristics of medical professional courses—specifically, the close integration of theory and practice and the emphasis on moral education. The scale comprises 13 items addressing core instructional behaviors, including classroom teaching design, interactive guidance, instructional support, and pedagogical integration. Representative items include “Incorporating interactive segments in class to encourage student participation (e.g., questioning, discussion),” “Providing case-based or example-driven discussions,” “Offering after-class tutoring and Q&A sessions for students,” “Integrating theoretical and practical instruction through learning-by-doing approaches,” and “Emphasizing moral education and the nurturing role of courses.” The scale demonstrated strong psychometric properties: Cronbach's α = 0.942, composite reliability (CR) = 0.945, and average variance extracted (AVE) = 0.57. Confirmatory factor analysis yielded the following fit indices: χ^2^/df = 8.917, NFI = 0.871, CFI = 0.887, and RMSEA = 0.136.

#### Professional identity evaluation scale

This scale measures medical students' cognitive, affective, and behavioral identification with the medical profession. It was adapted from dimensions related to professional attitude and career inclination in the aforementioned surveys, integrating theories of professional identity and competency requirements for medical professionals. The scale comprises 7 items assessing seven facets of professional identity: professional engagement, personality fit, learning interest, learning initiative, major reselection willingness, career commitment, and confidence in professional development. Representative items include “I frequently pay attention to industry hotspots and cutting-edge trends related to my major,” “My personality traits are well-suited to this major,” “I find the learning process of this major highly engaging,” “I would still choose this major if given the opportunity to reselect,” “I am willing to pursue a career aligned with my major,” and “I am confident in the future development of this major.” The scale demonstrated acceptable psychometric properties: Cronbach's α = 0.765, composite reliability (CR) = 0.782, and average variance extracted (AVE) = 0.346. Confirmatory factor analysis yielded the following fit indices: χ^2^/df = 6.684, NFI = 0.870, CFI = 0.886, and RMSEA = 0.115.

#### Self-reported professional competence scale

This scale assesses medical students' self-perceived development of professional literacy. Drawing upon dimensions related to core competency cultivation in the aforementioned research projects, it was developed in accordance with the core requirements for medical professionals' professional literacy—prioritizing medical ethics, emphasizing clinical competence, and pursuing all-round development. The scale comprises 11 items centered on the enhancement of professional literacy, encompassing eleven core dimensions: sense of responsibility, communication awareness, cost-effectiveness consciousness, learning orientation, respect for others, pragmatic attitude, innovative thinking, teamwork, legal awareness, patient-centered orientation, and critical thinking. The scale demonstrated excellent psychometric properties: Cronbach's α = 0.954, composite reliability (CR) = 0.957, and average variance extracted (AVE) = 0.668. Confirmatory factor analysis yielded the following fit indices: χ^2^/df = 12.327, NFI = 0.879, CFI = 0.887, and RMSEA = 0.162.

#### Campus environment satisfaction scale

This scale measures medical students' subjective satisfaction with the overall campus environment of their institution. Drawing upon dimensions related to campus environment and resource support in the aforementioned research projects [6, 7], it was developed in consideration of the distinctive characteristics of medical universities, which feature intensive experimental and practical training resources alongside rigorous academic discipline requirements. The scale comprises 11 items encompassing three dimensions: natural environment, soft environment, and hardware resources. Representative items include “Satisfaction with the campus natural environment,” “Satisfaction with the campus academic atmosphere and institutional culture,” “Satisfaction with campus classrooms and instructional equipment,” “Satisfaction with campus library resources (including electronic resources),” and “Satisfaction with campus laboratory and practical training facilities.” The scale demonstrated strong psychometric properties: Cronbach's α = 0.905, composite reliability (CR) = 0.912, and average variance extracted (AVE) = 0.485. Confirmatory factor analysis yielded the following fit indices: χ^2^/df = 7.018, NFI = 0.871, CFI = 0.887, and RMSEA = 0.118.

### Data processing

Descriptive statistics and Pearson correlation analyses were conducted using SPSS 27.0. To ensure analytical rigor, multicollinearity was assessed via the Variance Inflation Factor (VIF), with values exceeding 10 indicating severe multicollinearity warranting variable exclusion. Gender and academic year were included as covariates to control for potential demographic confounding.

Mediation and moderation analyses were performed using Model 4 and Model 1 of the Hayes PROCESS macro [33], respectively. The significance of indirect effects was tested using the bias-corrected percentile bootstrap method with 5,000 resamples. Effects were considered statistically significant when the 95% confidence interval excluded zero.

## Results

### Common method bias test

Harman's single-factor test in SPSS 27.0 showed that six principal components had eigenvalues greater than 1, and the variance explained by the first factor was 31.759%, which is below the 40% threshold, indicating that common method bias was not significant in this study.

Given that all data in this study were collected via self-reported questionnaires from medical students, there was a potential risk of common method bias. Thus, multiple rigorous tests were conducted to assess and rule out this issue. First, Harman's single-factor test was performed using SPSS 27.0. The results showed that six principal components had eigenvalues greater than 1, and the variance explained by the first factor was 31.759%, which was below the 40% threshold, initially indicating that common method bias was not significant in this study.

To further verify the robustness of the above result, confirmatory factor analysis (CFA) was conducted using AMOS to compare the model fit of three alternative models: a four-factor model (consisting of teaching behavior evaluation, professional identity evaluation, campus environment satisfaction, and self-reported professional competence), a two-factor model (combining teaching behavior evaluation with professional identity evaluation, and campus environment satisfaction with self-reported professional competence), and a single-factor model (all items loaded on one single factor). The results demonstrated that the four-factor model exhibited the best fit to the data (CMIN/DF = 3.104, RMSEA = 0.073, NFI = 0.806, CFI = 0.859), which was significantly superior to the two-factor model (CMIN/DF = 7.342, RMSEA = 0.122, NFI = 0.538, CFI = 0.572) and the single-factor model (CMIN/DF = 9.147, RMSEA = 0.138, NFI = 0.423, CFI = 0.422). Notably, all fit indices of the single-factor model were far below the acceptable criteria, indicating no single factor accounted for the majority of the variance in the measured variables.

Consistent results from Harman's single-factor test and CFA confirmed that common method bias was not a serious issue in the present study, and thus would not substantially affect the reliability and validity of the research findings.

### Descriptive statistics and correlation analysis of variables

Table 1 presents the descriptive statistics and Pearson correlation coefficients for all study variables. The results indicate significant positive correlations among students' evaluations of professional course instructors' teaching behaviors, professional identity, self-reported professional competence, and campus environment satisfaction. Regarding control variables, grade level was negatively correlated with professional identity and significantly negatively correlated with campus environment satisfaction, yet significantly positively correlated with self-reported professional competence and significantly negatively correlated with evaluations of professional course instructors' teaching behaviors. Gender was significantly negatively correlated with professional identity, but showed no significant associations with other key variables. These findings provide preliminary evidence for the intrinsic relationships among the core constructs under investigation and lay the groundwork for subsequent regression analyses.

**Table 1:** Correlation among variables (*N* = 430)

Item	Mean	Standard deviation	Gender	Grade	Professional Identity Evaluation	Campus EnvironmentSatisfaction	Self-Reported Professional Competence
Gender	1.64	0.481	1				
Grade	2.57	1.09	0.05	1			
Professional Identity Evaluation	3.973	0.496	−0.107*	−0.082	1		
Campus Environment Satisfaction	3.879	0.682	−0.004	−0.235**	0.232**	1	
Self-Reported Professional Competence	4.066	0.708	−0.042	0.215**	0.318**	0.275**	1
Teaching Behavior Evaluation of Professional Course Teachers	4.27	0.569	−0.058	-.098*	0.317**	0.496**	0.356**

Note: *p < 0.05, **p < 0.01

### Mediation association effect test

Model 4 from Hayes' SPSS PROCESS macro was used to test the mediating association role of professional identity evaluation in the relationship between teaching behavior evaluation and self-reported professional competence. The results (Tables 2 and 3) show that teaching behavior evaluation was significantly and positively associated with self-reported professional competence (β = 0.472, *p <* 0.001). After introducing the mediating variable, teaching behavior evaluation remained significantly and positively associated with self-reported professional competence; however, the correlation coefficient decreased significantly (β = 0.380, *p <* 0.001). Professional identity evaluation was significantly and positively associated with self-reported Professional Competence (β = 0.345, *p <* 0.001). Teaching behavior evaluation was significantly and positively associated with professional identity (β = 0.268, *p <* 0.001). Thus, the findings of this study are consistent with H1, H2, and H3. Table 3 presents the results for the total, direct, and indirect association effects. The total association effect was 0.472, the direct association effect was 0.380 (accounting for 80.51% of the total association effect), and the indirect association effect was 0.092 (accounting for 19.49%). The Bootstrap 95% confidence intervals for these results did not include zero, confirming the significance of the mediation association effect, thus the findings of this study are consistent with H4.

**Table 2:** Mediation model test of professional identity evaluation (*N* = 430)

Variable	Self-reported professional competence		Professional identity evaluation		Self-reported professional competence	
	β	t	β	t	β	t
Teaching Behavior Evaluation of Professional Course Teachers						
	0.472***	8.653	0.268***	6.685	0.380***	6.836
Professional Identity Evaluation					0.345***	5.401
*R*^2^	0.191		0.111		0.243	
*F*-value	33.593***		17.659***		34.155***	

Covariates of gender and grade level were included

^***^p < 0.001

**Table 3:** Total effect, mediating effect, and direct effect

	Effect value	Bootstrap 95% CI		Proportion of total effect
		Lower	Upper	
Total Effect	0.472	0.365	0.579	
Direct Effect	0.38	0.27	0.489	80.51%
Indirect Effect	0.092	0.047	0.149	19.49%

Covariates of gender and grade level were included

**Fig. 2:**
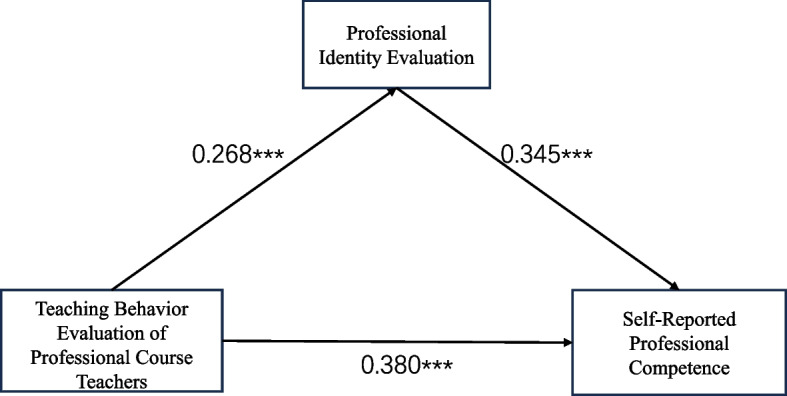
Mediation Model

The results indicate that teaching behavior evaluation is significantly and positively associated with self-reported professional competence, professional identity evaluation is significantly and positively associated with self-reported professional competence, and teaching behavior evaluation is positively associated with self-reported professional competence through the mediating association role of professional identity evaluation. The mediation model is illustrated in Fig. 2.

### Moderating association effect test

This study used Model 1 from Hayes' SPSS PROCESS macro to explore the relationship between professional identity evaluation and self-reported Professional Competence, examining the moderating association role of campus environment satisfaction as a moderator variable. Three models were established for in-depth analysis.

In each model, control variables (age and gender) were entered in the first step, followed by the predictor variables. Model 2 examined the relationship between professional identity evaluation and self-reported professional competence (the dependent variable), with gender and grade included as control variables to account for potential demographic confounds. Model 3 expanded upon Model 2 by adding campus environment satisfaction (the moderator) to assess its association with the main relationship between the independent and dependent variables. Model 4 further introduced the interaction term between professional identity evaluation and campus environment satisfaction to test their interactive effect on self-reported professional competence.

The results indicated that, after controlling for gender and grade, professional identity evaluation was significantly and positively associated with self-reported professional competence in Model 2 (β = 0.336, *p <* 0.001). Furthermore, the interaction term between professional identity evaluation and campus environment satisfaction demonstrated a significant positive association with self-reported professional competence in Model 4 (β = 0.087, *p <* 0.05). These findings suggest that campus environment satisfaction serves as a significant positive moderator of the relationship between professional identity evaluation and self-reported professional competence, and this moderating effect remains robust even after accounting for key demographic covariates. The results are presented in Table 4.

**Table 4:** Moderating effect test (*N* = 430)

Predictive variable	Self-reported professional competence			
	Model 1	Model 2	Model 3	Model 4
Gender	−0.053	−0.018	−0.027	−0.035
Grade	0.218***	0.244***	0.306***	0.300***
Professional Identity Evaluation		0.336***	0.275***	0.272***
Campus Environment Satisfaction			0.283***	0.292***
Professional Identity Evaluation × Campus Environment Satisfaction				0.087*
R^2^	0.049	0.160	0.232	0.239
Adjusted R^2^	0.045	0.154	0.225	0.230
F-value	11.039***	27.058***	32.094***	26.687***

Note: *p < 0.05, ***p < 0.001

**Fig. 3:**
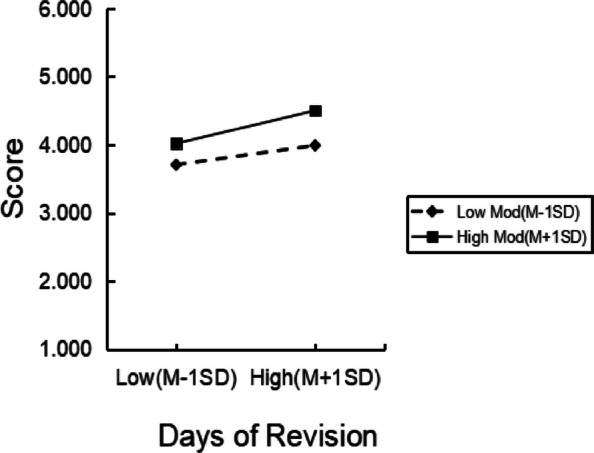
The moderating effect of high and low campus environment satisfaction

To examine whether the pattern of this moderating association effect was consistent with the hypothesis, we followed Aiken and West’s suggestions. Scores one standard deviation above the mean for campus environment satisfaction were considered high, and scores one standard deviation below the mean were considered low. The moderating association effects of different levels of campus environment satisfaction are shown in Table 5, and the simple slope plot is shown in Fig. 3. The positive correlation between professional identity evaluation and self-reported Professional Competence was stronger in the high campus environment satisfaction group and weaker in the low satisfaction group. Furthermore, at different levels of professional identity evaluation, the self-reported Professional Competence of the high-satisfaction group was higher than that of the low-satisfaction group. Thus, the findings of this study are consistent with H5.

**Table 5:** Moderating effect of campus environment satisfaction

Level of moderator	Effect	Standard error	t	95%CI	
Mean	0.388	0.062	6.204	0.265	0.511
Low Group (−1 SD)	0.285	0.082	3.492	0.125	0.445
High Group (+ 1 SD)	0.490	0.079	6.187	0.335	0.646

## Discussion

### Correlation between teaching behavior evaluation and professional identity evaluation

The finding of a positive correlation between professional course teachers' teaching behaviors and medical students' professional identity evaluation aligns with existing research in medical education. Empirical studies have demonstrated that clinical mentors' positive teaching behaviors during internships—such as instructional enthusiasm, a strong sense of responsibility, and exemplary role modeling—serve as critical determinants in shaping and enhancing medical students' professional identity [15]. This finding substantiates the central argument of the present study: teachers can directly facilitate students' internal acceptance of and value recognition toward the physician role through high-quality teaching interactions.

Nevertheless, the existing literature suggests that the influence of teaching behaviors may be embedded within a broader process of professional demonstration. Scholars have noted that medical students' evaluations of teachers' clinical practice behaviors—including passion for work and professional responsibility—may exert a more profound impact on their professional identity formation than assessments of purely pedagogical skills [34]. This observation offers a nuanced perspective for interpreting the present findings: teachers' verbal instruction (teaching behaviors) and behavioral demonstration (professional behaviors) collectively constitute the comprehensive environment influencing students' professional identity evaluation. Future research should further delineate the relative contributions of these two behavioral dimensions and examine their interaction effects.

From the perspective of students' perceived experiences, Valestrand et al. demonstrated that even the absence of fundamental interactive behaviors—such as greeting, addressing students by name, and maintaining eye contact—can precipitate professional alienation and self-doubt regarding suitability for the medical profession among clinical students [34]. This finding underscores the essential role of positive teaching interactions in constructing medical students' professional identity. Complementing this student-centered perspective, Sternszus et al., examining clinical teachers' self-perceptions, found that educators regard explicit role modeling, in-depth professional dialogue, and graded autonomy guidance as pivotal mechanisms for shaping students' professional identity [15]. Notably, these teachers perceived their professional behaviors in clinical practice as highly integrated with their teaching behaviors, forming an organic and cohesive demonstration of professionalism for students. Collectively, these studies have enriched the theoretical understanding of how teachers' behaviors influence medical students' professional identity evaluation and provide empirical justification for investigating the dual impact of teaching behaviors and professional behaviors on students' professional identity development.

### Correlation between professional identity evaluation and self-reported professional competence

This study reveals a significant positive correlation between medical students' professional identity evaluation and their self-reported professional competence. Specifically, stronger professional identity is associated with more favorable self-reported professional competence development. This core finding aligns closely with existing research in medical education and is robustly supported by empirical evidence across diverse populations and health professions.

Luthfianasari et al., in a study of 276 Indonesian medical students, demonstrated a moderate positive correlation between perceptions of professional identity and professionalism levels (r = 0.508, *p <* 0.001), corroborating the positive driving effect of professional identity on medical professional competence [35]. The underlying logic resonates with the present study: the value identification and emotional commitment engendered by professional identity motivate students to actively internalize professional standards and engage in clinical practice, thereby comprehensively enhancing their professional competence. Yao et al. further extended the generalizability of this conclusion in a study of 887 Chinese nursing interns, finding a significant positive correlation between professional identity and self-reported professional competence (r = 0.598, *p <* 0.01) and demonstrating that professional identity directly and positively predicted professional competence (β = 0.31, *p <* 0.01) [36]. These findings suggest that the "identity-competence" positive association represents a common pattern in health professions education.

Notably, Yao et al. elucidated the mediating role of self-efficacy in this relationship [36], with the indirect effect accounting for 52% of the total effect. This indicates that professional identity promotes students' active learning and practice by strengthening their vocational confidence, thereby offering a more nuanced mechanistic explanation for the direct "identity-competence" association observed in the present study. Concurrently, Luthfianasari et al. found that this positive association was not significantly moderated by study duration, further validating the generalizability of our findings [35].

In conclusion, professional identity serves as a critical intrinsic driving force for the development of medical students' self-reported professional competence—a conclusion that has been validated across different geographical regions and health-related disciplines. Future research should further explore the mechanisms of mediating variables such as self-efficacy to provide more targeted practical pathways for enhancing medical students' professional competence through strengthened professional identity, thereby contributing to the cultivation of high-quality medical professionals.

### Correlation between teaching behavior evaluation and self-reported professional competence

This study confirms a significant positive correlation between medical students' evaluation of professional course teachers' teaching behaviors and their self-reported professional competence. Specifically, higher student ratings of teachers' instructional performance are associated with more favorable self-reported professional competence development, whereas lower ratings may indicate a negative association. This core finding receives indirect support and logical corroboration from related research in medical education.

Quaintance et al. demonstrated that students' perceptions of faculty professional teaching behaviors—such as creating respectful learning environments and articulating clear professional standards—are strongly positively correlated with teachers' professional behavioral modeling [37]. Teaching behaviors inherently integrate knowledge transmission with professional demonstration; high student evaluations of such behaviors drive active imitative learning, thereby facilitating professional competence development. This mechanism indirectly validates the associative logic underlying the present study.

Spoto-Cannons et al., drawing on assessment data from 535 medical students, found that faculty ratings of students' clinical skills and professional competence were significantly higher than students' self-assessments, with no significant gender differences [38]. These results indicate that high-quality teaching behaviors translate into observable competence gains among students, while positive student evaluations of teaching behaviors are highly concordant with faculty recognition of student competence. This finding further substantiates the positive association between teaching behavior evaluation and professional competence development.

In conclusion, the quality of professional course teachers' teaching behaviors constitutes a critical factor influencing medical students' self-reported professional competence. Future research should incorporate the moderating role of professional atmosphere within the learning environment to further optimize teaching behavior assessment systems, thereby providing more targeted practical pathways for enhancing medical students' professional competence through improved instructional quality.

### The mediating association role of professional identity evaluation

This study confirms that there is a significant direct positive correlation between the evaluation of professional course teachers' teaching behaviors and medical students' self-reported professional competence, and an indirect promotion pathway is formed by enhancing students' professional identity. This core finding is supported and theoretically verified by relevant empirical research.

Quaintance et al. conducted a study on 371 medical students and 28 clinical teachers, revealing a strong positive correlation between students' perceptions of teachers' professional teaching behaviors (such as creating a respectful environment and clarifying professional standards) and teachers' professional behavioral modeling [37]. Essentially integrating knowledge transmission and professional demonstration, teachers' high-quality teaching behaviors enhance students' sense of professional belonging and identity, thereby driving students to engage in active practice and achieve professional competence improvement. This verifies the transmission logic of “teaching behaviors → professional identity → professional competence.”

Byram’s longitudinal qualitative research further reveals that teachers' teaching interactions, feedback, and exemplary role modeling are core triggering factors for the formation of medical students' professional identity [39]. Students deepen their professional identity through role imitation, value internalization, and participation in clinical practice, which ultimately translates into a significant improvement in self-reported professional competence, and this process occurs synchronously with the development of professional competence.

In summary, teachers' teaching behaviors serve as a key influencing factor in the development of medical students' professional competence through the dual pathways of directly imparting skills and indirectly strengthening identity. Future research can further quantify the relative contributions of these two pathways, integrate the moderating effect of the professional climate in the teaching environment, optimize the evaluation and intervention strategies of teachers' teaching behaviors, and provide more precise practical guidance for the professional training of medical talents.

### The moderating association role of campus environment satisfaction

This study identifies campus environment satisfaction as a critical moderating factor in the relationship between professional identity evaluation and medical students' self-reported professional competence, indicating that the strength of this association varies significantly across different levels of campus environment satisfaction. This core finding receives indirect empirical support and theoretical corroboration from relevant research.

Mukhalalati et al., in a mixed-methods study of 390 health professions students at Qatar University, confirmed a significant positive correlation between perceptions of the campus learning environment—encompassing core dimensions such as physical facilities, academic atmosphere, and teacher-student interactions—and professional identity development [40]. Their qualitative findings further revealed that a high-quality campus environment facilitates the translation of professional identity into professional competence by enhancing students' sense of professional belonging, whereas a suboptimal environment (e.g., inadequate facilities, excessive workload) attenuates this transformative effect. These findings substantiate the moderating mechanism of campus environment satisfaction.

Lai et al., examining 59 senior medical students in Malaysia, demonstrated a weak-to-moderate positive correlation between the campus learning environment and self-reported professional competence, with environment-sensitive dimensions such as “academic self-perception” exhibiting the strongest association with competence outcomes [41]. Although this study did not directly test the moderating effect, its findings suggest that the campus environment may indirectly alter the magnitude of professional identity's influence on competence by shaping students' learning confidence and engagement levels.

In conclusion, campus environment satisfaction modulates the relationship between professional identity and self-reported professional competence through dual mechanisms of enhancement and buffering. Future research should further quantify the differential moderating effects of specific environmental dimensions (e.g., physical infrastructure, academic climate), thereby providing more targeted practical pathways for enhancing medical students' professional competence through strategic optimization of the campus environment and strengthened professional identity development.

## Conclusion

Based on social cognitive theory and ecological systems theory, this study systematically examined the mechanisms linking professional course teachers' teaching behavior evaluation to medical students' self-reported professional competence, with a particular focus on the mediating role of professional identity evaluation and the moderating effect of campus environment satisfaction. The principal findings are as follows: (1) Evaluation of professional course teachers' teaching behavior demonstrated a significant positive association with medical students' professional identity evaluation. (2) Professional identity evaluation exhibited a significant positive association with medical students' self-reported professional competence. (3) Evaluation of professional course teachers' teaching behavior showed a significant positive association with students' self-reported professional competence. (4) Professional identity evaluation partially mediated the relationship between teaching behavior evaluation and self-reported professional competence. (5) Campus environment satisfaction significantly moderated the relationship between professional identity evaluation and self-reported professional competence, such that higher levels of campus environment satisfaction substantially strengthened the positive association between professional identity and self-reported professional competence.

The theoretical contribution of this study lies in revealing the association path of “teacher teaching behavior–professional identity–self-reported Professional Competence” and incorporating campus environment satisfaction into the analysis framework of moderating association effects, thereby expanding the theoretical research boundary on the correlation between teacher teaching behavior and medical students' career development. The research results provide the following insights for universities to optimize teaching strategies and promote the cultivation of medical students' self-reported Professional Competence: First, efforts should be made to improve the teaching quality of professional course teachers. Universities can promote teachers to improve teaching methods and enhance professional demonstration through teacher training, teaching competitions, and incentive mechanisms, thereby being associated with the improvement of medical students' professional identity and self-reported Professional Competence. Second, the cultivation of medical students' professional identity should be emphasized. Universities can help medical students deepen their understanding and passion for their profession by optimizing the curriculum, strengthening professional cognitive education, and providing practical opportunities, which further shapes their positive self-reported Professional Competence. Furthermore, medical students should actively participate in professional practice and career exploration to strengthen their professional identity through action. Finally, the campus environment should be optimized continuously. Universities can enhance medical students' campus environment satisfaction by improving hardware facilities, creating a positive and uplifting campus cultural atmosphere, providing diversified support services, and creating favorable conditions for the correlation between professional identity and self-reported Professional Competence.

In summary, by revealing the association mechanism between professional course teachers' teaching behavior evaluation, professional identity evaluation, campus environment satisfaction, and self-reported Professional Competence, this study provides a theoretical basis and practical reference path for universities to cultivate high-quality medical professionals. Future research could further explore the differences in the above association mechanisms across different disciplinary backgrounds and types of institutions, and combine subjective self-evaluation with objective professional competence indicators to deepen the understanding of the relationship between teachers’ teaching behavior and medical students' actual professional competence, providing more scientific evidence for the quality improvement and sustainable development of higher medical education.

### Research limitations

This study has several limitations that should be acknowledged when interpreting the findings. First, the study adopted a cross-sectional research design with a single-wave questionnaire survey, and all core variables were measured by self-report of medical students at the same time point. This design only verifies the statistical correlation between variables and cannot determine the temporal order of the variables, thus failing to make causal inferences about the relationship between teaching behavior evaluation, professional identity evaluation, campus environment satisfaction and self-reported Professional Competence. It is theoretically plausible that there is a reverse or reciprocal correlation between variables—for example, medical students with a higher level of self-reported Professional Competence or professional identity may rate teachers' teaching behaviors and campus environment more favorably. Moreover, the core outcome variable of this study is self-reported Professional Competence, which is measured by students' subjective evaluation rather than objective professional competence indicators (e.g., Objective Structured Clinical Examination (OSCE) scores, supervisor-rated clinical skills, standardized academic achievements). This measurement method may lead to self-enhancement bias and social desirability bias—medical students may overestimate their professional competence due to positive self-perception, or provide overly positive self-evaluations to meet social and educational expectations, which may affect the objectivity of the correlation results between variables. Second, the research sample was only selected from a single medical university in Guizhou Province, which may limit the generalizability of the findings to other medical universities or regions in China. Future research can expand the sample size and select multi-center samples to improve the external validity of the research results. Third, the follow-up research can adopt a longitudinal research design to track the dynamic changes of medical students' teaching behavior evaluation, professional identity and self-reported Professional Competence over time, so as to further explore the potential causal relationship between variables. Fourth, future research can combine subjective self-reported evaluation with objective professional competence indicators to conduct mixed-method studies, so as to more accurately reveal the relationship between teacher teaching behavior evaluation and medical students' actual professional competence.

## Supplementary Information

Supplementary Material 1.

## Data Availability

The data that support the findings of this study are available from the corresponding author, Zhongxian Duan, upon reasonable request.
